# A next generation targeted amplicon sequencing method to screen for insecticide resistance mutations in *Aedes aegypti* populations reveals a *rdl* mutation in mosquitoes from Cabo Verde

**DOI:** 10.1371/journal.pntd.0010935

**Published:** 2022-12-13

**Authors:** Emma L. Collins, Jody E. Phelan, Magdalena Hubner, Anton Spadar, Monica Campos, Daniel Ward, Holly Acford-Palmer, Ana Rita Gomes, Keily Silva, Lara Ferrero Gomez, Taane G. Clark, Susana Campino

**Affiliations:** 1 Faculty of Infectious and Tropical Diseases, London School of Hygiene and Tropical Medicine, London, United Kingdom; 2 Laboratory of Pathogen-Host Interactions (LPHI), CNRS, Montpellier University, Montpellier, France; 3 Universidade Jean Piaget (UniPiaget), Praia, Cabo Verde; 4 Faculty of Epidemiology and Population Health, London School of Hygiene and Tropical Medicine, London, United Kingdom; QIMR Berghofer Medical Research Institute, AUSTRALIA

## Abstract

*Aedes* mosquito vectors transmit many viruses of global health concern, including dengue, chikungunya and Zika. These vector-borne viral diseases have a limited number of treatment options, and vaccines vary in their effectiveness. Consequently, integrated vector management is a primary strategy for disease control. However, the increasing emergence and spread of insecticide resistance is threatening the efficacy of vector control methods. Identifying mutations associated with resistance in vector populations is important to monitor the occurrence and evolution of insecticide resistance and inform control strategies. Rapid and cost-effective genome sequencing approaches are urgently needed. Here we present an adaptable targeted amplicon approach for cost-effective implementation within next generation sequencing platforms. This approach can identify single nucleotide polymorphisms (SNPs) and small insertions and deletions (indels) in genes involved in insecticide resistance in *Aedes aegypti* mosquitoes. We designed and tested eleven amplicons, which included segments of the *ace-1* (carbamate target), the *Voltage-Gated Sodium Channel* (*vgsc*; pyrethroids, DDT and organochlorines), and *rdl* (dieldrin) genes; thereby covering established knockdown resistance (kdr) mutations (e.g., S989P, I1011M/V, V1016G/I and F1534C), with the potential to identify novel ones. The amplicon assays were designed with internal barcodes, to facilitate multiplexing of large numbers of mosquitoes at low cost, and were sequenced using an Illumina platform. Our approach was evaluated on 152 *Ae*. *aegypti* mosquitoes collected in Cabo Verde, an archipelago with a history of arbovirus outbreaks. The amplicon sequence data revealed 146 SNPs, including four non-synonymous polymorphisms in the *vgsc* gene, one in *ace-1* and the 296S *rdl* mutation previously associated with resistance to organochlorines. The 296S *rdl* mutation was identified in 98% of mosquitoes screened, consistent with the past use of an organochlorine compound (e.g., DDT). Overall, our work shows that targeted amplicon sequencing is a rapid, robust, and cost-effective tool that can be used to perform high throughput monitoring of insecticide resistance.

## Author summary

Many viruses, such as dengue and Zika, are transmitted by *Aedes aegypti* mosquitoes. These vector-borne diseases are a major public health problem in the tropics and sub-tropics worldwide. The primary strategy to reduce their burden is vector control, including through the application of insecticides. However, many mosquito populations have developed resistance to insecticides. Here, we present a rapid, robust, and cost-effective tool that allows the screening of genes associated with insecticide resistance across many *Aedes aegypti* mosquitoes, identifying known resistance and novel mutations. This assay will support vector control strategies by informing on the emergence and spread of insecticide resistance mutations across *Aedes aegypti* populations.

## Introduction

Vector-borne diseases pose a major risk to public health, causing ~700k deaths every year [1]. Arthropod-borne viruses (arboviruses), causing dengue, Zika and chikungunya infections, contribute substantially to the global burden of disease. Mosquitoes of the genus *Aedes* are responsible for the transmission of many arboviruses, with *Ae*. *aegypti* being one of the most competent vectors. The distribution of *Ae*. *aegypti* has increased dramatically in recent years, predominantly due to their adaptation to urban environments and the globalization of human activities [2,3]. Vector control strategies are essential to prevent arboviral spread, largely due to the lack of effective vaccines and available antiviral drugs. Vector control predominantly involves the use of insecticides, either in the form of spraying or treated bed nets [4,5]. However, the intensive use of insecticides worldwide has led to the emergence of resistance to pyrethroids, organochlorines, carbamates, neonicotinoids and organophosphates [6,7], which is threatening the effectiveness of vector control campaigns for important vector-borne diseases.

The main mechanisms of insecticide resistance are target site, metabolic and cuticular, and behavioural avoidance [6,8]. Target site resistance is caused by point mutations in genes that encode the protein targeted by the insecticide, including voltage gated sodium channels (*vgsc* gene), acetylcholinesterase (*ace-1*) and the γ-aminobutyric acid (GABA) receptor (resistance to dieldrin locus *rdl*) [9]. VGSC proteins are present in the nervous system and are a target for DDT and pyrethroids. Knockdown resistance (kdr) to these two insecticides has been linked to multiple target site mutations in the *vgsc* gene in many insects [9]. Acetylcholinesterase (AChE) enzymes hydrolyse the neurotransmitter acetylcholine at the synaptic cleft and hence terminate nerve signals. Organophosphates and carbamate insecticides bind to AChE thus disrupting nerve impulses and ultimately causing death. A single target site mutation in the *ace-1* gene (G119S), encoding AChE, has been shown to inhibit the insecticidal action in many mosquito vectors [10,11] including *Ae*. *aegypi* [12]. Finally, mutations in the *rdl* gene (e.g. A301S *Drosophila melanogaster*, A296 in many mosquito species including *Ae*. *aegypti*, *Ae*. *albopictus and Anopheles arabiensis* [13] *and* V327I *An*. *funestus* [14]), have been associated with resistance to organochlorine insecticides in *Anopheles*, *Aedes* and *Culex* vectors [15–17].

Current methods for the identification of insecticide resistance involve biological and biochemical assays [18–20] which are time-consuming, require multiple repeats, and involve subjective judgement of mosquito knockdown. Additionally, bioassays can often only detect resistance when frequencies are already high, and molecular methods may be required if resistant alleles are at lower frequencies [21,22]. Molecular methods have been developed for the detection of mutations associated with insecticide resistance and can be an effective approach to monitor resistant alleles when diagnostic markers predictive of vector control intervention failure are known [10,23–25]. Given the recent innovations and cost reductions in molecular techniques, testing based on the molecular underpinning of insecticide resistance is likely to be an effective approach to support monitoring. This innovation would allow public health organizations to monitor the emergence and spread of known resistance mutations and detect the appearance of new genetic polymorphisms. In addition, alongside biological and biochemical assays of susceptibility, molecular surveillance can identify novel resistance markers and provide insights into the mechanisms of action.

Amplicon sequencing is a targeted next-generation sequencing method that allows for the high throughput detection of low frequency variants in specific genomic regions of interest. Here we describe a multiplexed amplicon sequencing approach targeting the *vgsc*, *ace-1* and *rdl* loci of *Ae*. *aegypti* mosquitoes. The assays target eleven genomic regions across these three genes where mutations associated with insecticide resistance have been reported in *Aedes* and other vectors. A dual index approach was used with an individual barcoding system that allows for the pooling and simultaneous sequencing of multiple PCR products. Sequence data are later demultiplexed to individual mosquitoes and genes from raw sequence data, providing a fast and cost-effective surveillance method to detect mutations involved in insecticide resistance. To demonstrate the utility of our approach, it was applied to *Ae*. *aegypti* mosquitoes from Cabo Verde, an archipelago located 500 kilometres off the coast of West Africa. Dengue and Zika outbreaks have been reported in Cabo Verde [26–28]. In 2009, more than 21,000 cases of dengue fever were diagnosed and in 2015 an epidemic of Zika caused at least 7,580 reported cases. To prevent vector-borne disease, Cabo Verde has a history of applying several strategies to combat *Anopheles*, *Aedes* and *Culex* vectors, including the past use of DDT (organochlorine) and recent spraying of temephos (organophosphate) and deltamethrin (pyrethroid) insecticides [29]. Compared to *Anopheles* mosquitoes, little is known about *Aedes* insecticide resistance and associated mutations, in both Cabo Verde, and in Africa as a whole [30]. VGSC mutations (e.g., V1016I, F1534C) that confer resistance to pyrethroids have been reported at low frequency in Cabo Verde [26] but no other mutations were investigated. Using the dual index amplicon-based approach on an Illumina sequencing platform, we screen for known mutations associated with insecticide resistance in *Ae*. *aegypti* sourced from Cabo Verde. Through this work, we demonstrate the utility of our approach for detecting insecticide resistance mutations as well as novel polymorphism, to inform vector-borne disease control efforts.

## METHODS

### Amplicon primer design

A list of target site insecticide resistance mutations in *Aedes* vectors was extracted from an OVID search and recent reviews [9,31–41]. Overall, twelve mutations linked to insecticide resistance were found, nine in the *vgsc* (V410L, G923V, L982W, S989P, I1011V/M, V1016I/G, T1520I, F1534C/L, D1763Y), one in *rdl* (A301S), and one in *ace-1* (G119S). Sequences for *vgsc* (AAEL023266-RL), *ace-1* (AAEL000511-RJ) and *rdl* (AAEL008354-RA) were extracted from publicly available assemblies for *Ae*. *aegypti* (LVP AGWG). The mutations of interest were identified by performing a BLAST sequence alignment of the *Ae*. *aegypti* reference genome against the sequences of species in which the mutation had been described (see S1 Table).

A summary of the amplicon approach is provided (S1 Fig). Forward and reverse primers were designed using PrimerBLAST software to amplify regions of 450-500bp that contained known SNP loci or regions of interest. The primers ranged from 18-25bp in length and were designed to have similar annealing conditions to allow for multiplexing (Table 1). Primers targeting eleven regions were selected across three genes. Primers were checked for cross-hybridization in each multiplex combination using ThermoFisher Multiplex Primer Analyser. Each multiplex PCR used a combination of at least 3 targets (Table 1). To allow pooling of individual mosquito PCR products, a 6bp barcode was added to the 5’ end of each primer (forward and reverse unique barcodes) to distinguish individual mosquito products after sequencing (S2 Table). To each mosquito DNA sample, forward and reverse 6bp barcodes were assigned across all loci, allowing the pooling of many different mosquito PCR products. Partial Illumina tails of ~30bp were also added to the 5’ end of each primer, just after the 6bp barcode primer-tag, for compatibility with commercial sequencing. This feature allows the pools to be sequenced using any Illumina sequencer, in-house or by commercial providers. A second PCR carried out by the commercial sequencing company enabled the addition of Illumina adaptors and indexes if necessary to pool experiments (S1 Fig).

**Table 1:** Amplicon regions, mutations previously described and primer sequences.

Gene	Primer Name	Mutations	Primer Sequence (5’-3’)*	Amplicon Length	Multiplex
*vgsc*	DomainI	V410L	F: [TTTCGTCTAATGACCCAAGA] R: [ARAGAWTTCGCTCACCCG]	464	1
*vgsc*	DomainIIIExon35	T1520I, F1534C/L	F: [GGATCCAGATCATGAACGAY] R: [GATGATCATGTCGAACTTCT]	480	1
*Rdl*	Rdl_Aeg	A301S	F: [CCAACCGATGTATCTTCTTC] R: [CTGGTTATTTGTACAAGTAGCA]	498	1
*Ace-1*	Ace1	G119S	F: [TCGCYTRGCCGAAGCCGT] R: [CASGTGAARTGATAATCTCCSAC]	468	1
*vgsc*	DomainIIS4	G923V	F: [TCTAGATTTAGYGACTCCAR] R: [TACCGATGTAGTTCTTGCC]	444	2
*vgsc*	DomainII	L982W, S989P, I1011V/M, V1016I/G	F: [ACTCRTTCATGATCGTGTTC] R: [GACTTGATCCAGTTGGAGA]	498	2
*vgsc*	DomainIIIExon36	NA	F: [GTGTCATCATCGACAACTTC] R: [CACACCTAAAATGGACAGGA]	489	2
*vgsc*	DomainIV	D1763Y	F: [GCGATCTSATCGAGAAGTA] R: [ATGCTAGCAARTACGTGATG]	495	2
*vgsc*	DomainIIExon26	982W, S989P, I1011V/M	F: [TCACCTTATGCTAAGACTTCA] R: [GGGAAACAATTTGTCGGTTA]	494	3
*vgsc*	DomainIIIExon33_34	T1520I, F1534C/L	F: [AACTCTCTATTCCCGCTTG] R: [GCAGATCATTCGTAACAAGT]	469	3
*vgsc*	Domain IVS6	NA	F: [TGTTGGACGGTATCATCAA] R: [CCTCGATCGGRTTACCTTT]	456	3

*Primer sequences underlined show incompatible nucleotides with *Aedes albopictus* reference sequence (FOSHAN).

### Mosquito collection, DNA extraction, PCR, and purification

A pooled sample of *Ae*. *aegypti* was used as control to test the amplicon sequencing primers. *Ae*. *aegypti* mosquitoes were collected in the city of Praia, on Santiago island in Cabo Verde [42]. The mosquitoes were all morphologically identified as *Ae*. *aegypti*. Mosquito DNA was extracted according to the manufacturer’s instructions for Qiagen DNeasy Blood and Tissue kits. DNA concentration was determined using Qubit. Multiplex PCR was carried out under the conditions: Initial denaturation (98.0°C, 30 seconds) followed by 35 cycles of denaturation (98.0°C, 10 seconds), annealing (60.4°C, 70 seconds), and extension (72.0°C, 90 seconds). Each reaction comprised reagents from Q5 High-Fidelity PCR kit (New England Biolabs, UK), 4μl of DNA template and 0.5 μl of each forward and reverse primer at 10pmol/μl. Three multiplex PCRs were carried out (Table 1). PCR products were visualised on a SYBR safe (Cambridge Bioscience,UK) 1% agarose gel alongside a 100bp ladder. PCR products were purified with AMPure XP magnetic beads (Beckman Coulter), using a ratio of 0.8:1 (μl of beads to DNA).

### Sequencing and bioinformatics analysis

The DNA concentration of purified PCR products was tested with the Qubit 2.0 fluorimeter HS DNA kit (ThermoFisher, Waltham, MA, USA). DNA concentrations varied between 7.9 and 47.7 ng/μl. All PCRs were diluted and grouped in equal concentrations to create an overall pool of 20 ng/μl in 25 μl total volume, containing around 220 amplicons (11 amplicons across 20 mosquitoes per pool = 220 amplicons). A second PCR to insert IIlumina adaptors and indexes to pool experiments (no further library preparation is required), which allows many pools to be sequenced in the same Illumina run. This second PCR step, followed by amplicon sequencing, was performed by Genewiz (from Azenta Life Sciences) at a cost of ~US$ 60 per pool (~220 amplicons, US$ 0.30 per amplicon). A minimum of 50,000 reads (250bp read pairs) were obtained per pool, equivalent to an average of ~220 reads per amplicon. From the sequenced pool, individual mosquito data were demultiplexed based on the 6bp barcode primer-tag in each forward and reverse primer using an inhouse pipeline (https://github.com/LSHTMPathogenSeqLab/amplicon-seq), which removed any mis-tagging across barcodes. Sequences were trimmed and aligned to the *Ae*. *aegypti* reference (LVP AGWG). Sequence data were checked for quality using FastQC (v 0.11.5). Paired end reads were mapped against the reference sequence using the BWA-MEM algorithm (v0.7.17, default parameters). SNPs and small indels were called using freebayes (v1.3.5,—haplotype-length -1) and GATK HaplotypeCaller (v 4.1.4.1, default parameters) software tools. Variants detected across either software caller were used as an initial set for characterisation across amplicons. High quality SNPs were identified using filters that included a minimum phred quality score of 30 per called base, a minimum depth of 50 reads, and a minimum allele depth of 10-fold. Only SNPs that were present in more than one mosquito, and present across two independent pools were retained. The bioinformatics pipeline is summarised (S2 Fig). The distribution of allele depth and frequency for each SNP was analysed to assign threshold cut-offs for genotyping calls as described for diploid organisms [43]. The annotation of the SNP identified was called using bcftools csq (v1.1.0, default parameters). Sanger sequencing using individual forward or reverse primers, was performed for 38 mosquitoes to confirm the findings of the amplicon sequencing for the *ace* and *rdl* amplicon regions. Chi squared tests were performed to assess possible deviations from the Hardy-Weinberg Equilibrium. Tajima’s D test was applied to distinguish between sequences evolving randomly ("neutrally") and evolving under a non-random process (e.g., selection, demographic expansion/contraction). This test was implemented using MEGA11 software [44].

## Results

### Target amplicon representation and variant calling

**Fig 1:**
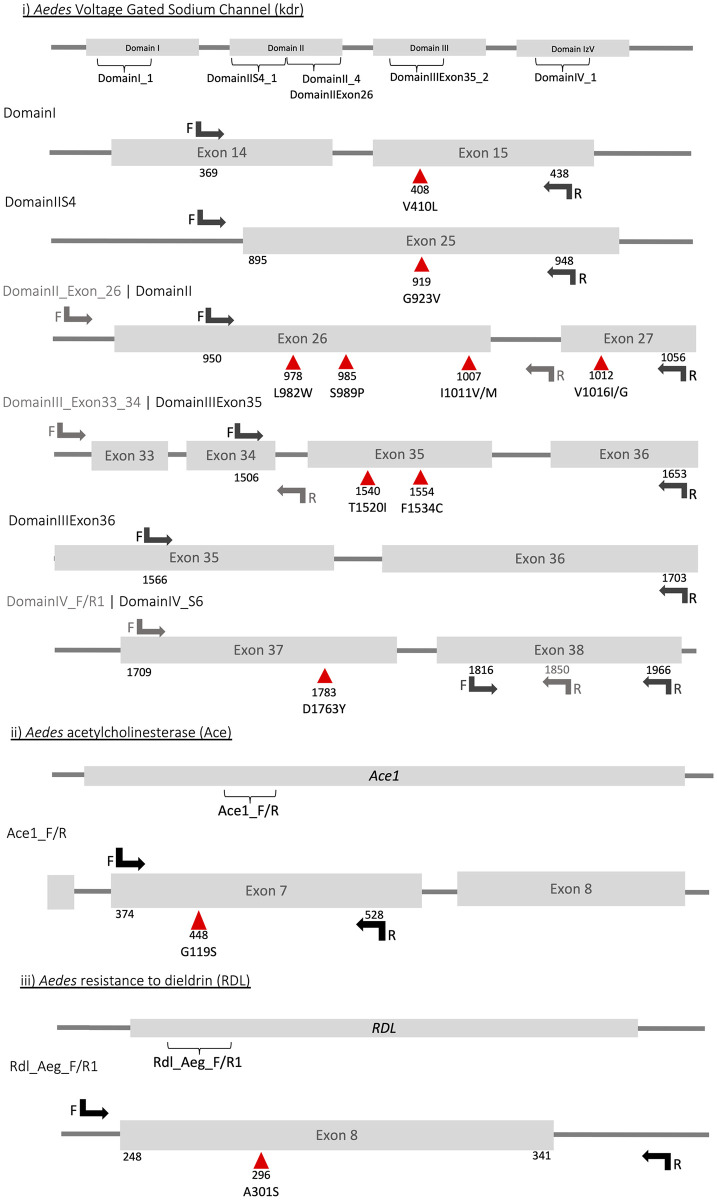
Aedes mutations and primer positions for *ace-1*, *vgsc* and *rdl* genes. Previously described mutations associated with insecticide resistance are represented with red triangles and primers are shown with arrows. The first and last codon that are amplified are under each amplicon diagram.

A total of eleven ~500bp amplicon assays were designed across *vgsc* (nine amplicons spanning the positions of nine known insecticide mutations), *ace-1* (1 amplicon; 1 known mutation) and *rdl* (1 amplicon; 1 known mutation) loci (see Fig 1). Each amplicon assay was validated individually and across multiplex PCRs, and it was possible to amplify the eleven loci multiplexed in three different PCR reactions (Table 1).

A total of 152 *Ae*. *aegypti* mosquitoes sourced from Cabo Verde were processed individually using the multi-locus amplicon assays. For each mosquito, 11 amplicons were obtained containing the same combination of barcodes (forward and reverse 6bp barcode primer-tag) (S2 Table). A unique combination was used for each mosquito across the 11 amplicons in each pool. Each pool consisted of 220 amplicons (11 loci across 20 mosquitoes) and was sequenced on an Illumina platform. This number was selected to obtain a high coverage per amplicon, with a minimum of 50,000 reads per pool sequenced (average 220 reads per amplicon) being obtained (See Methods). A bioinformatics pipeline was developed to demultiplex from the pools each mosquito data (using 6bp barcode primer-tag) from raw sequencing data, remove mistagging sequences, perform alignment to reference strain, call variants and genotypes, whilst removing low-quality data and variants (see Methods). The pipeline revealed some minor differences in genomic coverage, reflecting differences in the amplification of regions, due to the expected differences in the efficiency of primer binding. Overall, coverage varied between amplicons with DomainIIExon26Aeg (*vgsc* gene) having the lowest mean coverage of 120-fold while DomainIIIExon36 (*vgsc* gene) had nearly 20 times higher-fold coverage. The average read count over all amplicons was 936-fold (Table 2).

**Table 2:** The eleven amplicons and their sequencing performance in 152 Cabo Verde *Ae*. *aegypti*.

Locus	Domain	Exon	known mutations [observed]	Amplicon Length	Mean read depth before filtering	No. SNPs * (Exonic)
*ace-1*	-	-	1 [0]	468	669	3 (3)
*vgsc*	I	-	1 [0]	464	328	24 (0)
*vgsc*	II	-	4 [0]	498	137	25 (2)
*vgsc*	II	26	3 [0]	494	120	27(3)
*vgsc*	III	33_34	0 [0]	469	1389	26 (6)
*vgsc*	III	35	2 [0]	480	2289	38 (15)
*vgsc*	III	36	0 [0]	489	2352	32 (13)
*vgsc*	II	S4	1 [0]	444	191	7 (1)
*vgsc*	IV	-	1 [0]	495	516	6 (3)
*vgsc*	IV	S6	0 [0]	456	613	10 (10)
*rdl*	-	-	1 [1]	498	418	1 (1)
***Total***	-	-	14 (9 unique)	**-**	-	240 (72) (146 unique)

*Amplicon regions overlap therefore SNPs are found in multiple amplicons

A total of 146 SNP variants were identified. To validate our pipeline, 87 *Ae*. *aegypti* individual mosquitoes were re-sequenced and added to different pools, leading to all SNPs being confirmed and a concordance of 84.2% obtained for genotype calls. The genotype differences were observed at only five positions, between homozygous and heterozygous calls, and only in the mosquitoes with allele ratios of 0.8–1.0, where one allele in all tested mosquitoes and replicates is present in the majority of total reads.

Further, Sanger sequencing was performed for 38 mosquitoes to confirm the findings of the amplicon sequencing for the *ace* and *rdl a*mplicon regions. A 90% concordance in genotyping calls between Sanger sequencing and amplicon sequencing was observed for the *ace* amplicon. For the only SNP detected in the *rdl* gene a 67% genotype concordance was observed between the two methods. Again, discordant genotype calls were observed in heterozygous mosquitoes using amplicon sequencing that were homozygous with Sanger sequencing, and these heterozygous mosquitoes had an allele ratio close to 0.8, showing an increase of one allele in the total reads. It is possible that mistagging rearrangement, as previously highlighted [45,46], could lead to an unexpected distribution of allele frequencies across mosquitoes and differences in genotype calls, particularly leading to excess heterozygous genotypes. By assigning threshold cut-offs based on allele ratios as described for other diploid organisms [43] we can identify and reassign the most likely genotype. Therefore, by recalling heterozygous genotypes with an allele ratio from 0.8–1 into homozygous, a 100% genotype concordance was obtained. The allele frequency spectrum across all genes reveals an excess of low frequency alleles (~40% of SNPS with minor allele frequency (MAF) < 0.1) close to neutrality (Tajima D’ = -0.56; close to zero). There are some distortions from HWE (37% of exonic and 43% of intronic SNPs with P < 0.001) (Table 3), which could be the result of the amplicon method leading to an excess of heterozygous. These results need to be further investigated in larger studies, and by including more mosquito generations.

**Table 3:** Position and frequency of synonymous and non-synonymous SNPs in each gene for *Ae*. *aegypti*.

Chromosome	Exon	Position	Reference allele	Alternative allele	Consequence	Number	Alternative Allele Frequency (%)
3	Exon 7	161500198	A	C	466L > 466V	105	1
3	Exon 26	315984096	C	T	977V > 977L	80	51.4*
3	Exon 35	315939050	G	T	1612P > 1612H	147	12.2
3	Exon 35	315939101	T	G	1595N > 1595T	139	9
3	Exon 35	315939155	T	G	1577K > 1577T	106	0.9
2	Exon 8	41847790	G	T	296A > 296S	68	62.5*
3	Exon 7	161500076	T	A	Synonymous	108	100
3	Exon 7	161500262	G	A	Synonymous	104	29.3*
3	Exon 26	315984075	G	A	Synonymous	88	10.3
3	Exon 26	315984159	C	T	Synonymous	38	40.8*
3	Exon 34	315939469	G	A	Synonymous	108	2.8
3	Exon 34	315939517	G	T	Synonymous	107	3.3
3	Exon 34	315939547	G	A	Synonymous	112	7.6
3	Exon 33	315939648	C	T	Synonymous	105	27.1*
3	Exon 35	315939229	G	A	Synonymous	140	10.4
3	Exon 35	315939241	A	G	Synonymous	137	71.9*
3	Exon 35	315939244	G	A	Synonymous	138	1.8
3	Exon 35	315939274	A	G	Synonymous	139	1.8
3	Exon 35	315939283	C	T	Synonymous	131	11.5
3	Exon 34	315939367	C	A	Synonymous	148	10.5*
3	Exon 34	315939373	G	A	Synonymous	149	0.3
3	Exon 36	315938745	G	A	Synonymous	83	53.4*
3	Exon 36	315938760	A	G	Synonymous	78	72.8*
3	Exon 36	315938772	C	T	Synonymous	80	3.6
3	Exon 36	315938775	C	T	Synonymous	69	28.3*
3	Exon 36	315938778	C	T	Synonymous	66	43.2*
3	Exon 36	315938832	C	T	Synonymous	75	2.7
3	Exon 36	315938946	A	C	Synonymous	141	0.4
3	Exon 35	315939088	C	T	Synonymous	130	1.5
3	Exon 35	315939112	T	C	Synonymous	110	73.6*
3	Exon 35	315939166	C	T	Synonymous	108	55.1*
3	Exon 25	315998391	C	T	Synonymous	60	3
3	Exon 37	315932072	G	A	Synonymous	95	16.7
3	Exon 37	315932142	A	G	Synonymous	96	42.1*
3	Exon 37	315932184	G	A	Synonymous	92	72.9*
3	Exon 38	315931422	G	A	Synonymous	96	12
3	Exon 38	315931428	C	T	Synonymous	97	14.4
3	Exon 38	315931440	T	C	Synonymous	95	5.3
3	Exon 38	315931470	T	C	Synonymous	94	14.9*
3	Exon 38	315931479	C	A	Synonymous	97	3.6
3	Exon 38	315931485	G	A	Synonymous	95	5.3
3	Exon 38	315931557	T	C	Synonymous	97	57.7*
3	Exon 38	315931563	T	C	Synonymous	95	16.3*
3	Exon 38	315931575	G	A	Synonymous	94	4.8
3	Exon 38	315931578	G	A	Synonymous	93	11.3

* Significant deviation from HWE (p<0.001)

### SNPs and insecticide resistance variants

**Fig 2:**
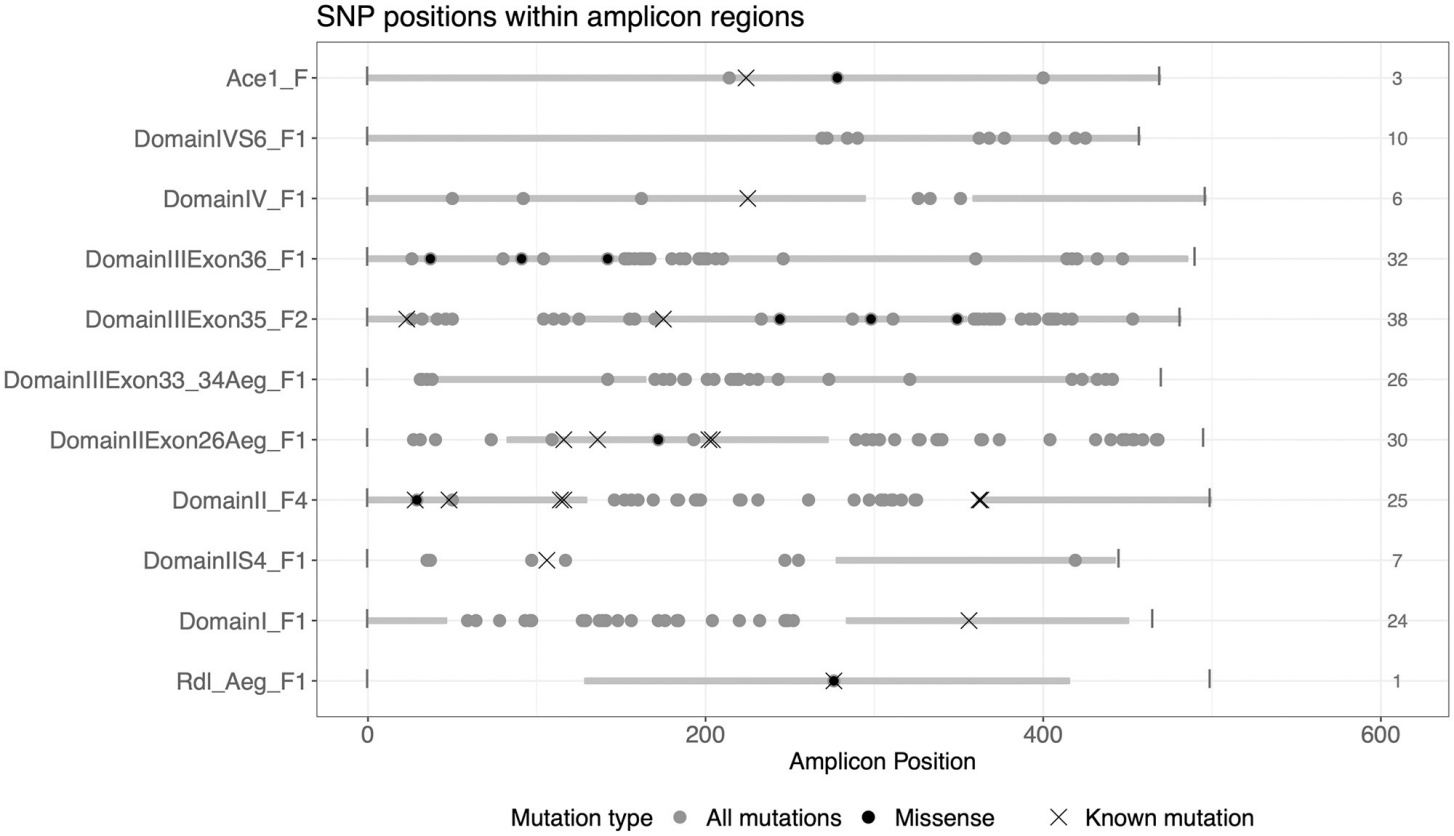
Distribution of SNPs detected in the eleven amplicons. Grey shaded bars illustrate exonic regions. Black points show the position of missense mutations identified, grey points are other SNPs identified, and crosses mark where known mutations associated with insecticide resistance are positioned. The value on the right is the number of SNPs identified in each amplicon.

The analysis pipeline detected 146 SNP variants of which 45 were exonic. The number of SNPs identified in each region was highly variable, with the majority in the *vgsc* Exon 35 amplicon (38 SNPs, 11 exonic) and the least in the *rdl* amplicon (1 exonic SNP) (Table 3 and Fig 2). Almost all exonic SNPs led to synonymous changes (39 SNPs), and six led to missense genetic polymorphisms. Thirteen SNPs occurred in predicted splice regions, determined using the bcftools csq tool. Polymorphisms in splice regions may have little effect on gene function, but can alter the splicing pattern, such as skipping an exon or keeping of large segments of an intron in the mRNA, which can affect the protein function [47]. Further functional studies will be needed to confirm the *in silico* predictions of SNPs in splice regions.

The highest number of polymorphisms was found in the *vgsc* gene, largely due to the higher number of primers (9 of 11 amplicons) targeting these loci, with 142 SNPs identified. Mutations in the *vgsc* gene or *ace* gene previously associated with insecticide resistance (S1 Table) were not identified. Four amino-acid substitutions (V977L, K1577T, N1595T, P1612H) were found in *vgsc* gene. Of interest is the substitution V977L as it is next to a known mutation, L978W, (homologous to position L982W in *Drosophila melanogaster*), which is reported to confer resistance to pyrethroids [48,49].

In the *rdl* amplicon, we identified the amino acid substitution A296S, known to be associated with resistance to organochlorines [33]. The A296S mutation (analogous to position 301 in *D*. *melanogaster*) was identified in 47 mosquitoes (47/48; 70.1% heterozygous; 27.1% mutant homozygous). In the *ace-1* amplicon, three SNPs were found, two being synonymous (T506T, D444D) and one missense translation (L466V). For the T506T mutation (161500076 T>A; n = 108), which has been previously reported in Indonesian mosquitoes [50], all mosquitoes were homozygous for the non-reference allele. The L466V amino acid change was found at a low frequency (1/105, 0.95%; 100% heterozygous). The known G119S insecticide resistance mutation (position G448S in *Ae*. *aegypti*) was not detected in any of the mosquitoes investigated here.

## Discussion

Insecticide resistance is a threat to vector control programs worldwide. Traditional methods to identify resistance can be subjective and time-consuming, therefore molecular surveillance is becoming an attractive option to determine the widespread distribution of insecticide resistance and to complement diagnostic bioassays. Our study successfully demonstrates that multi-locus target amplicon sequencing can be used to identify insecticide resistance polymorphisms in *Ae*. *aegypti* mosquitoes in a robust, cost-effective, and high-throughput manner. By testing the approach on *Ae*. *aegypti* mosquitoes from the city of Praia in Santiago, Cabo Verde, it was possible to identify 146 SNPs across *vgsc*, *rdl*, and *ace-1* loci, including the *rdl* A301S mutation linked to organochlorine insecticide resistance. This observation is probably due to the past use of an organochlorine (e.g., DDT) in Cabo Verde. Resistance to this insecticide has been reported to have the least effect on fitness compared to other pyrethroid resistant populations [51]. No other known polymorphisms associated with insecticide resistance were detected. A previous study in Santiago, with samples collected between 2017 and 2018, detected the kdr polymorphism 1016I in two heterozygous individuals from São Lourenço dos Órgãos, and the 1534C mutation at low frequency (≤ 2.0%) in mosquitoes from Praia, but these variants were not observed in previous years (2007 to 2016) [26,42,52]. These results suggest a recent origin of kdr mutations in this island, that could be an independent event or an introduction from neighbouring countries of mainland West Africa. Cabo Verde is located ~500 kilometres off the coast of Senegal, where none of these mutations have been identified in a survey performed in 2017 [53]. These mutations were also not identified in Cameroon, Congo and Central African Republic, but were reported in Ghana and Burkina Faso in high frequency [54–57].

We also have identified further polymorphisms, the majority in intronic regions, some in predicted splice regions or leading to synonymous changes, and only five non-synonymous amino acid substitutions in the *ace-1* and *vgsc* genes. For example, the substitution V977L in the *vgsc* gene which is next to the previously reported L978W (position L982W in *D*. *melanogaster*) reported to confer resistance to pyrethroids [48,49]. There is also the synonymous variant in the *ace-1* gene (T506T in *Ae*. *aegypti* reference), which has been described in an Indonesian *Ae*. *aegypti* temephos resistant population [50], and could be in high linkage disequilibrium with a functional polymorphism, but these results have not been confirmed in other studies.

Identification and exploration of these additional polymorphisms can be useful to understand the evolution of these loci and their possible involvement in mechanisms of insecticide resistance, including through linkage with other polymorphisms or *cis*-regulatory elements and the generation of alternative transcripts. Further genotype-phenotype studies will be fundamental to explore the possible involvement of these mutations in insecticide resistance.

More generally, investigations of insecticide resistance in the *Aedes* population have largely focused on *kdr* variations in the *vgsc* gene, with little focus on the other loci investigated here. Molecular testing for insecticide resistance has been limited across West Africa and the wider continent, with only a few studies focusing on a limited number of polymorphisms, typically using a genotyping approach. For instance, the recent studies performed in Senegal, Cameroon, Ghana, Cabo Verde and Ivory Coast [42,53–55,58] focused only on the study of the kdr mutations F1534C, V410L, V1016G/I and S989P in *Ae*. *aegypti*. More surveys are necessary across the *vgsc* gene and other loci to understand the frequency, emergence and spread of genetic variants and their association with insecticide resistance.

As demonstrated here, the multi-locus amplicon approach gives the possibility to inform on both known and discover novel genetic variants in many loci simultaneously and across large numbers of samples. Examining both known and novel SNPs is highly valuable due to the large unexplained variance observed in insecticide resistance. The assays are adaptable and can be extended to include new loci linked to resistance. For example, the current panel does not account for metabolic mechanisms of resistance, which involve the upregulation of detoxifying enzymes in the mosquito, such as cytochrome P450. Only a few SNPs have been associated with this type of resistance and the underlying genes involved remain unclear, but our assays can be extended with further understanding. Sequence capture followed by deep sequencing has been applied to *Ae*. *aegypti* to investigate copy number variations (CNVs) and polymorphisms of detoxification enzymes [59]. However, this system requires the production of capture libraries with overlapping RNA probes, becoming a more expensive and complicated approach than using multiplex PCR assays. Quantitative PCR, like the one developed by Cattel *et al* [60], is still a main approach for the rapid detection and copy number quantification in *Ae*. *Aegypti*.

The multi-locus PCR amplicon approach can be applied across many loci and it is possible to pool large numbers of samples which can be differentiated by unique barcode combinations. Further, there is no need to prepare libraries for Illumina sequencing, as the PCR stages already include Illumina-compatible flow-cell adaptor sequences, leading to multi-locus amplicon pools that can be sequenced by commercial providers at relatively low cost (<$0.50 USD per amplicon). The same amplicons can also be sequenced using portable sequencers (e.g., Oxford Nanopore MinIon), leading to more rapid and informative surveillance of insecticide resistance at field sites, and an improved response to emerging resistance. Relatedly, there is the potential to pool mosquitoes before the PCR step, for rapid overall population surveillance, as opposed to individual mosquito amplification as performed here [21]. This facilitates application to lower income settings, where *Aedes* borne diseases are endemic, and the benefits of informed vector control will be greater. Specifically, the identification of important insecticide resistance mutations would provide early warning and evidence for the need to change the insecticide class prior to total inefficacy.

The main limitation of our amplicon approach is the prior need of information concerning which loci are associated with insecticide resistance. There is no substitute for insecticide bioassays to determine phenotype response. The multi-locus amplicon approach can be used to complement bioassays, and obtain genotyping data alongside phenotypic information, to inform functional work seeking to understand the molecular mechanisms underlying insecticide resistance. Our assay does not currently detect the genomic contributors of metabolic resistance; however, our approach is highly flexible, and further regions of interest can be added to the panel where required. The design of the methodology to target regions where mutations are likely to occur is also beneficial as it means that some mutations that are newly discovered are already captured in the panel. For example, a new mutation (A1007G) was recently described in *Ae*. *aegypti* to be putatively associated with DDT resistance, and is already captured by the DomainII_F4 amplicon, although it was not detected in our mosquitoes [61]. Moreover, there is the possibility to include pathogen screening in parallel with insecticide resistance characterisation to allow for monitoring of arboviruses and other pathogens in an integrated surveillance programme. It has already been demonstrated that it is possible to detect the malaria parasites from human blood using amplicon sequencing [62,63].

Overall, our work outlines a cost-effective, robust, and high-throughput methodology to screen *Ae*. *aegypti* mosquitoes for both known and putative novel insecticide resistance mutations. The integration of 5’ tag barcodes allow the pooling of many mosquitoes and loci within applications of next-generation sequencing, and their subsequent separation during analysis. These molecular and bioinformatic approaches can be implemented by vector control programs to monitor insecticide resistance and improve the efficacy of other approaches. The extension of the approach to other genomics regions, pathogens and other vectors will further assist with supporting control strategies of vector-borne diseases and reducing their global burden.

## Supporting information

S1 Fig Steps for multiplex amplicon sequencing. In the first PCR, target genes are amplified and partial Illumina tails and 6bp barcodes included in primers to differentiate individual samples. In a second step the amplicons are pooled across samples. After, a second PCR is performed in each pool, the Illumina adapters and indexes are added, and pools are ready to be sequenced using an Illumina platform.
(TIF)

S2 Fig Flow chart of the bioinformatics pipeline. (TIF)

S1 Table Amino acid mutation positions for the reference organism and corresponding *Ae*. *aegypti*. (DOCX)

S2 Table The 6bp barcodes and partial Illumina tails added to the 5’ end of the forward and reverse primers. (DOCX)

S3 Table Details of the SNPs detected in splice regions. (DOCX)
