# Supplementary figures and images for: A next generation targeted amplicon sequencing method to screen for insecticide resistance mutations in Aedes aegypti populations reveals a rdl mutation in mosquitoes from Cabo Verde
Source: PLoS Negl Trop Dis. 2022 Dec 13;16(12):e0010935. doi: 10.1371/journal.pntd.0010935 (PMC9746995; doi:10.1371/journal.pntd.0010935)

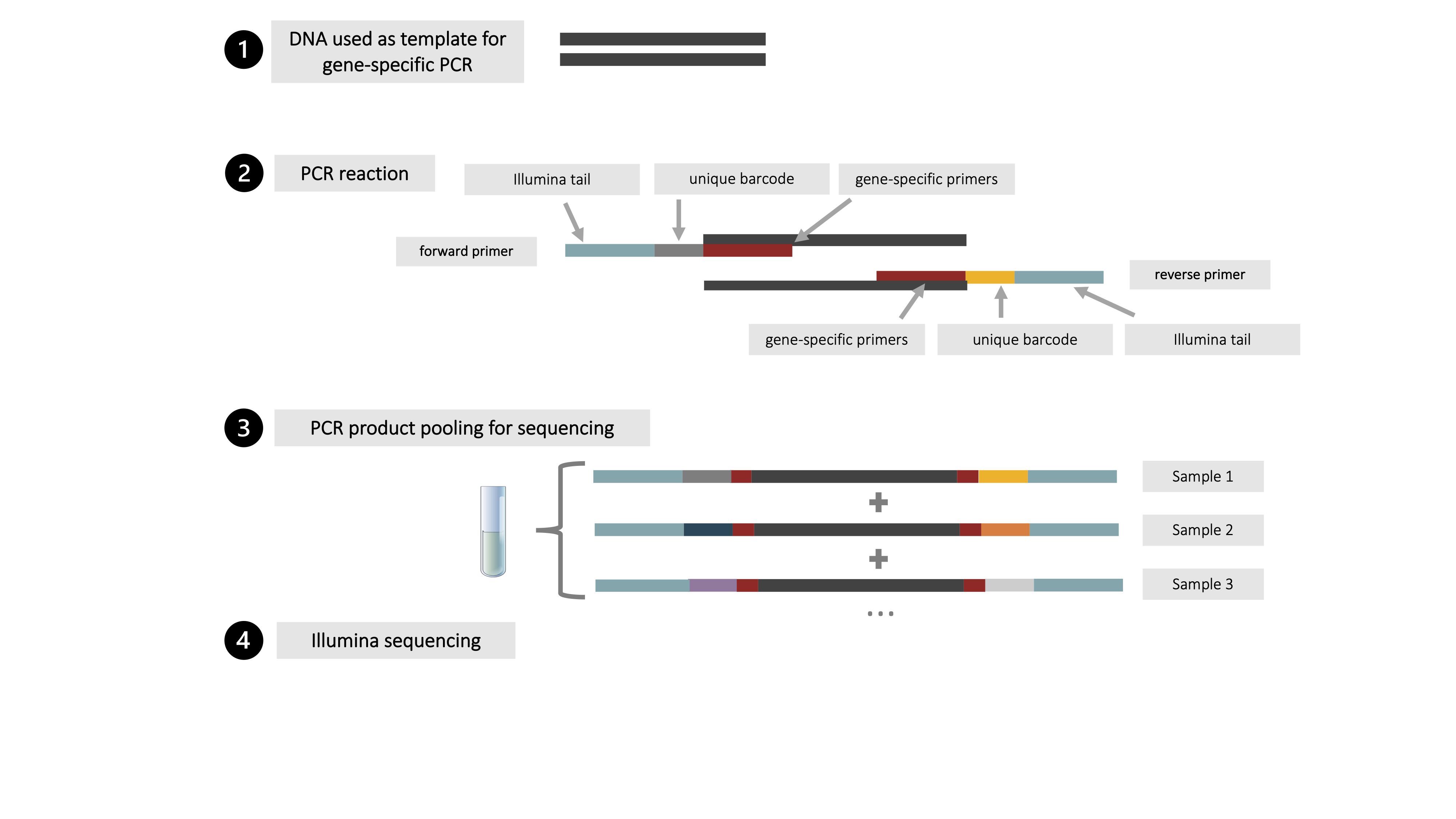

Supplement: S1 Fig — In the first PCR, target genes are amplified and partial Illumina tails and 6bp barcodes included in primers to differentiate individual samples. In a second step the amplicons are pooled across samples. After, a second PCR is performed in each pool, the Illumina adapters and indexes are added, and pools are ready to be sequenced using an Illumina platform. (TIF) [file pntd.0010935.s001.tif]

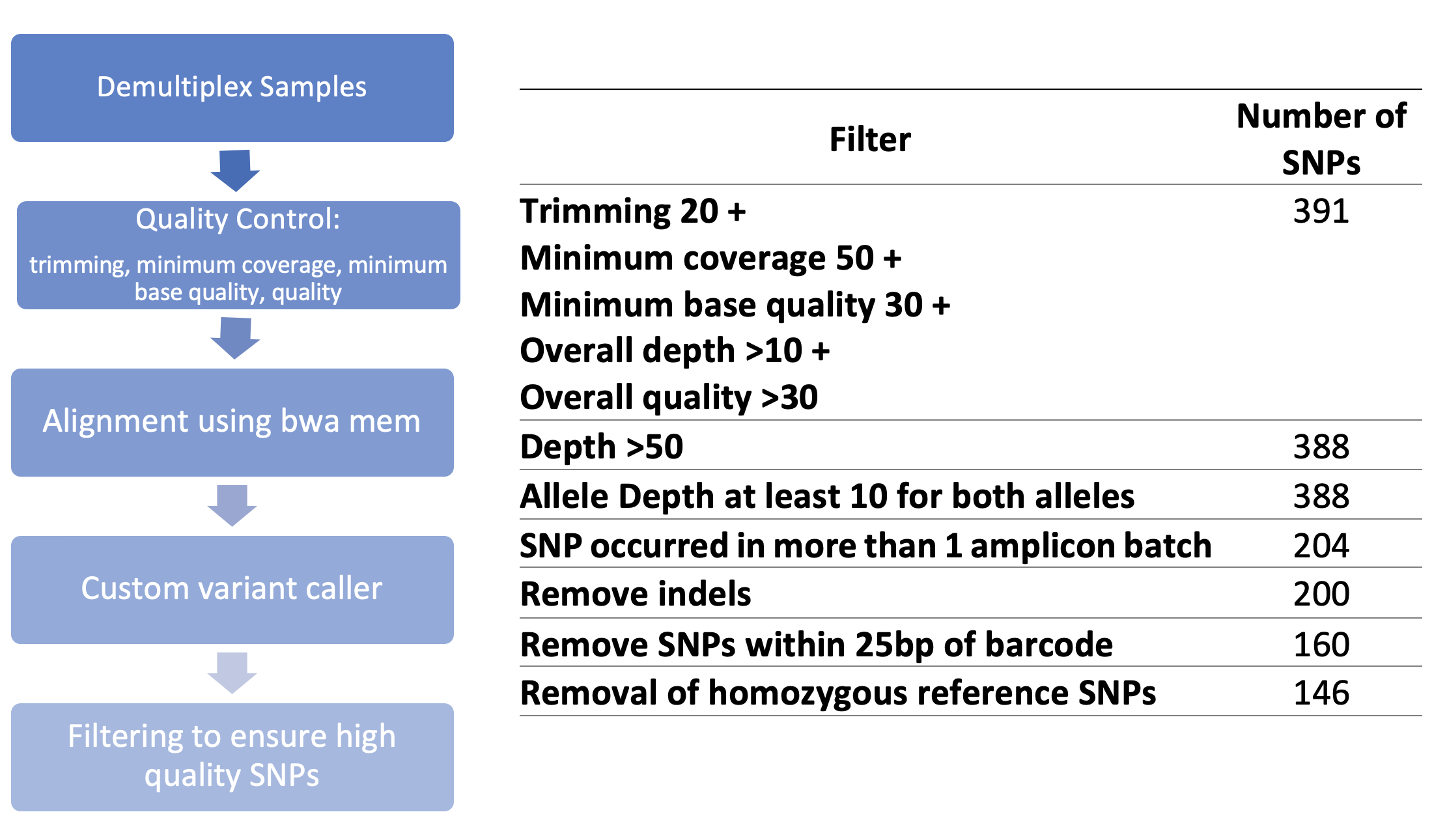

Supplement: S2 Fig — (TIF) [file pntd.0010935.s002.tif]
