# Supplementary material for: A next generation targeted amplicon sequencing method to screen for insecticide resistance mutations in Aedes aegypti populations reveals a rdl mutation in mosquitoes from Cabo Verde
Source: PLoS Negl Trop Dis. 2022 Dec 13;16(12):e0010935. doi: 10.1371/journal.pntd.0010935 (PMC9746995; doi:10.1371/journal.pntd.0010935)
Supplement: S1 Table — (DOCX) [file pntd.0010935.s003.docx]

**Supplementary Table 1.** Amino acid mutation positions for the reference organism and corresponding *Ae. aegypti.*

| **Gene** | **Mutation Position Reference** | **Reference Species** | **Mutation Position**  ***Ae. aegypti*** |
| --- | --- | --- | --- |
| *vgsc* | V410L | *Musca domestica* | 419 |
| *vgsc* | G923V | *Musca domestica* | 919 |
| *vgsc* | L982W | *Musca domestica* | 978 |
| *vgsc* | S989P | *Musca domestica* | 985 |
| *vgsc* | I1011V/M | *Musca domestica* | 1007 |
| *vgsc* | V1016I/G | *Musca domestica* | 1012 |
| *vgsc* | T1520I | *Musca domestica* | 1540 |
| *vgsc* | F1534C/L | *Musca domestica* | 1554 |
| *vgsc* | D1763Y | *Musca domestica* | 1783 |
| *rdl* | A301S | *Drosophila melanogaster* | 296 |
| *Ace-1* | G119S | *Torpedo californica* | 448 |
