# Supplementary material for: A next generation targeted amplicon sequencing method to screen for insecticide resistance mutations in Aedes aegypti populations reveals a rdl mutation in mosquitoes from Cabo Verde
Source: PLoS Negl Trop Dis. 2022 Dec 13;16(12):e0010935. doi: 10.1371/journal.pntd.0010935 (PMC9746995; doi:10.1371/journal.pntd.0010935)
Supplement: S2 Table — (DOCX) [file pntd.0010935.s004.docx]

**Supplementary Table 2.** DNA in-line barcodes and Illumina platform tails added to the 5’ end of the forward and reverse primers before PCR amplification

| **Forward Barcode Name** | **Illumina tail** | **Barcode** |
| --- | --- | --- |
| B1 | ACACTCTTTCCCTACACGACFCTCTTCCGATCT | ATCACG |
| B2 | ACACTCTTTCCCTACACGACFCTCTTCCGATCT | CGATGT |
| B3 | ACACTCTTTCCCTACACGACFCTCTTCCGATCT | TTAGGC |
| B4 | ACACTCTTTCCCTACACGACFCTCTTCCGATCT | TGACCA |
| B5 | ACACTCTTTCCCTACACGACFCTCTTCCGATCT | ACATGT |
| B6 | ACACTCTTTCCCTACACGACFCTCTTCCGATCT | TGCCAA |
| B7 | ACACTCTTTCCCTACACGACFCTCTTCCGATCT | AGCTCG |
| B8 | ACACTCTTTCCCTACACGACFCTCTTCCGATCT | ACGTCA |
| B9 | ACACTCTTTCCCTACACGACFCTCTTCCGATCT | GCAGAT |
| B10 | ACACTCTTTCCCTACACGACFCTCTTCCGATCT | GATCAC |
| **Reverse Barcode Name** | **Illumina tail** | **Barcode** |
| BR1 | ACACTCTTTCCCTACACGACFCTCTTCCGATCT | CAGATC |
| BR2 | ACACTCTTTCCCTACACGACFCTCTTCCGATCT | ACTTGA |
| BR3 | ACACTCTTTCCCTACACGACFCTCTTCCGATCT | GATCAG |
| BR4 | ACACTCTTTCCCTACACGACFCTCTTCCGATCT | TAGCTT |
| BR5 | ACACTCTTTCCCTACACGACFCTCTTCCGATCT | GGCTAG |
| BR6 | ACACTCTTTCCCTACACGACFCTCTTCCGATCT | CTTGTA |
| BR7 | ACACTCTTTCCCTACACGACFCTCTTCCGATCT | TGAGAT |
| BR8 | ACACTCTTTCCCTACACGACFCTCTTCCGATCT | ATGTGC |
| BR9 | ACACTCTTTCCCTACACGACFCTCTTCCGATCT | GTATCA |
| BR10 | ACACTCTTTCCCTACACGACFCTCTTCCGATCT | CTACTG |
