# Supplementary material for: A next generation targeted amplicon sequencing method to screen for insecticide resistance mutations in Aedes aegypti populations reveals a rdl mutation in mosquitoes from Cabo Verde
Source: PLoS Negl Trop Dis. 2022 Dec 13;16(12):e0010935. doi: 10.1371/journal.pntd.0010935 (PMC9746995; doi:10.1371/journal.pntd.0010935)
Supplement: S3 Table — (DOCX) [file pntd.0010935.s005.docx]

**Supplementary Table 3. Details of the SNPs detected in putative splice regions.**

| **Position** | **Reference** | **Alleles** | **Total samples** |
| --- | --- | --- | --- |
| 315938982 | A | G | 146 |
| 315938986 | T | G | 144 |
| 315939038 | T | C | 148 |
| 315939039 | G | T | 146 |
| 315939040 | C | T | 147 |
| 315939289 | C | A | 138 |
| 315939295 | G | A | 134 |
| 315939353 | T | A | 151 |
| 315939358 | T | G | 149 |
| 315939559 | A | C | 110 |
| 315939620 | C | T | 107 |
| 315939752 | A | G | 106 |
| 315939755 | T | G | 108 |
